# Human metabolic chambers reveal a coordinated metabolic-physiologic response to nutrition

**DOI:** 10.1172/jci.insight.184279

**Published:** 2024-11-22

**Authors:** Andrew S. Perry, Paolo Piaggi, Shi Huang, Matthew Nayor, Jane Freedman, Kari E. North, Jennifer E. Below, Clary B. Clish, Venkatesh L. Murthy, Jonathan Krakoff, Ravi V. Shah

**Affiliations:** 1 Vanderbilt Translational and Clinical Cardiovascular Research Center, Vanderbilt University School of Medicine, Nashville, Tennessee, USA.; 2 Phoenix Epidemiology and Clinical Research Branch, National Institute of Diabetes and Digestive and Kidney Diseases, NIH, Phoenix, Arizona, USA.; 3 Department of Biostatistics, Vanderbilt University School of Medicine, Nashville, Tennessee, USA.; 4 Sections of Cardiovascular Medicine and Preventive Medicine and Epidemiology, Department of Medicine, Boston University School of Medicine, Boston, Massachusetts, USA.; 5 CVD Genetic Epidemiology Computational Laboratory, Gillings School of Global Public Health, University of North Carolina, Chapel Hill, North Carolina, USA.; 6 Vanderbilt Genetics Institute, Division of Genetic Medicine, Department of Medicine, Vanderbilt University Medical Center, Nashville, Tennessee, USA.; 7 Broad Institute of MIT and Harvard, Cambridge, Massachusetts USA.; 8 Department of Medicine, University of Michigan, Ann Arbor, Michigan, USA.

**Keywords:** Metabolism, Amino acid metabolism, Carbohydrate metabolism, Intermediary metabolism

## Abstract

Human studies linking metabolism with organism-wide physiologic function have been challenged by confounding, adherence, and precision. Here, we united physiologic and molecular phenotypes of metabolism during controlled dietary intervention to understand integrated metabolic-physiologic responses to nutrition. In an inpatient study of individuals who underwent serial 24-hour metabolic chamber experiments (indirect calorimetry) and metabolite profiling, we mapped a human metabolome onto substrate oxidation rates and energy expenditure across up to 7 dietary conditions (energy balance, fasting, multiple 200% caloric excess overfeeding of varying fat, protein, and carbohydrate composition). Diets exhibiting greater fat oxidation (e.g., fasting, high-fat) were associated with changes in metabolites within pathways of mitochondrial β-oxidation, ketogenesis, adipose tissue fatty acid liberation, and/or multiple anapleurotic substrates for tricarboxylic acid cycle flux, with inverse associations for diets with greater carbohydrate availability. Changes in each of these metabolite classes were strongly related to 24-hour respiratory quotient (RQ) and substrate oxidation rates (e.g., acylcarnitines related to lower 24-hour RQ and higher 24-hour lipid oxidation), underscoring links between substrate availability, physiology, and metabolism in humans. Physiologic responses to diet determined by gold-standard human metabolic chambers are strongly coordinated with biologically consistent, interconnected metabolic pathways encoded in the metabolome.

## Abstract 2

Broad metabolomic profiling during controlled dietary pertubations in metabolic chambers reveals mechanisms of human metabolism.

## Introduction

Interindividual responses to a nutritional challenge have been important to specifying heterogeneity in metabolism relevant to health (1). Many human studies have employed prolonged fasting, caloric restriction (2–5), or shorter- versus longer-term responses to macronutrient feeding (6–10) to start to discern potential contributors to this metabolic heterogeneity (human genetics, resting metabolic rate, substrate oxidation, thermogenesis; refs. 3, 11–13). This work builds on the long history of those and many other studies that have contributed to the understanding of the physiologic, endocrine, and metabolic responses to these interventions (reviewed in refs. 14, 15). Despite all the previous work in this area, in-depth studies of how an individual’s metabolism responds to dietary challenge remain difficult, challenged by adherence to prescribed caloric and macronutrient content during free living, variability in other measures of metabolism during dietary challenge (e.g., activity, weight, sleep), and lack of accurate and complete measures (over 24 hours versus just resting) of substrate oxidation preference and energy expenditure (EE) traits, which are variable across individuals (3, 16, 17). Akin to studies in model systems, whole-room indirect calorimetry in humans has emerged in response to these limitations, allowing precise control over nutrition, confounding (activity, sleep), and weight to assess key physiologic responses to nutrition (13, 18). Nevertheless, human chamber studies are time and cost intensive and have traditionally not measured molecular metabolic states in humans across different dietary exposures, critical to understanding the complex interplay between diet and human metabolism.

Here, we address this gap in a longitudinal, approximately 40-day inpatient crossover study including 97 unique participants during which participants were fed highly controlled weight-maintaining diets (WMDs) and had 7 monitored, 24-hour diets with different macronutrient composition in a whole-room indirect calorimeter. Our effort was aimed at building a set of reference data in a highly controlled setting to study the effect of dietary intervention on heterogeneity in metabolism. We addressed this aim by mapping molecular changes in a circulating metabolome onto gold-standard substrate oxidation and EE traits (defined by precise minute-by-minute measures over 24-hour). Our modeling approach took advantage of the unique crossover study design to provide insight into coordinated metabolic-physiologic responses within an individual, linking excursions in metabolites to macronutrient processing via 24-hour EE and oxidation profiles for lipid, carbohydrate, and protein. Our goal was to provide human data in the largest-to-date investigation of metabolite profiling, to our knowledge, during dietary challenge in human metabolic chambers to link substrate availability, oxidation, EE, and metabolism.

## Results

### Study design and participant physiologic characterization.

**Figure 1:**
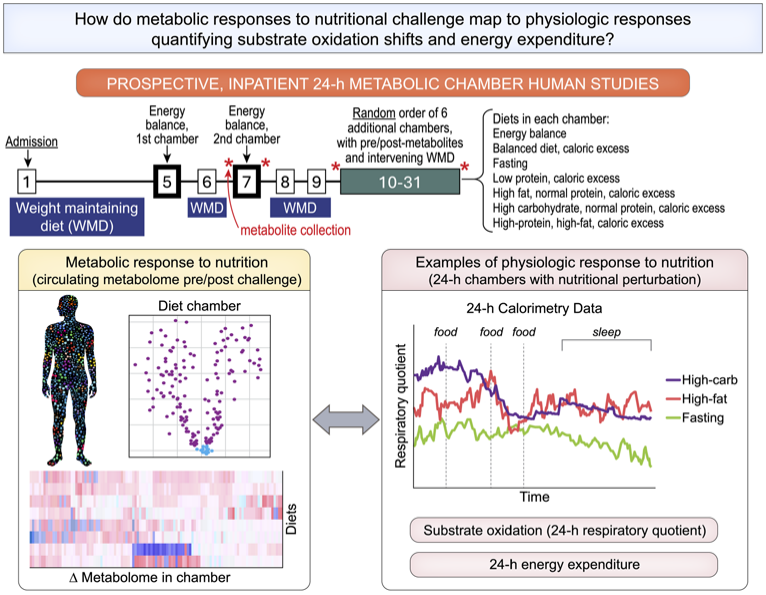
Experimental design. Study scheme, detailing inpatient clinical research protocol and study aims.

Figure 1 shows our experimental design. Nutritional interventions (with macronutrient composition in Figure 1 and Supplemental Table 1; supplemental material available online with this article; https://doi.org/10.1172/jci.insight.184279DS1) included an “energy balance” chamber (to determine an individual’s caloric needs at balanced intake = expenditure), fasting, and serial diets with differing macronutrient composition with 200% caloric excess relative to energy balance. The 200% caloric excess was used as a physiologic probe to elicit phenotypes defined by 24-hour EE and 24-hour substrate oxidation rate (13). Dietary intervention chambers after energy balance were performed in random order with an intervening 3-day washout period, during which participants remained domiciled and consumed a WMD. We used indirect calorimetry during chambers to quantify 24-hour EE and substrate oxidation preferences (24-hour respiratory quotient [RQ], lipid, carbohydrate, and protein oxidation rate), as described (13). This design allowed efficient, precise control over confounding (e.g., activity level, sleep, diurnal effects, weight) to quantify phenotypes that capture interindividual heterogeneity in metabolism.

**Figure 2:**
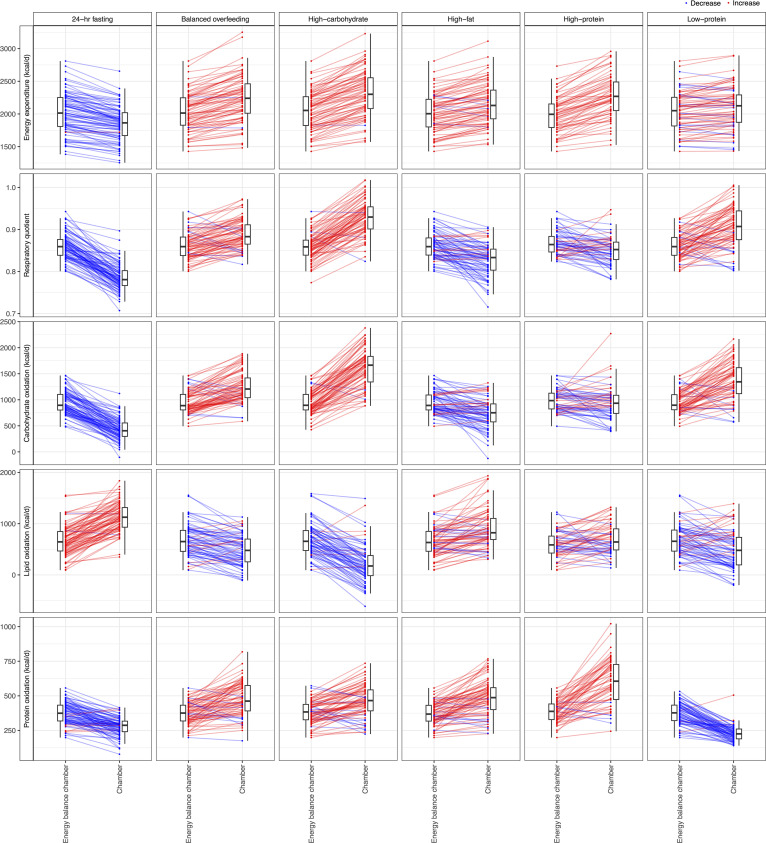
Heterogeneity in metabolic responses to dietary perturbation. Here, we present changes in 5 metabolic parameters (rows) from all dietary chambers, with comparison to the energy balance chamber. Bars represent the mean change across all participants, and points represent individual participants. While most participants followed the average trend, some individuals displayed opposing changes (e.g., in the high-fat chamber, the average change in respiratory quotient was a decrease; however, in some participants, the respiratory quotient increased). Red indicates that the average effect was an increase in the metabolic parameter during the dietary chamber compared with energy balance; blue indicates a decrease.

The characteristics of our study group are shown in Table 1. Overall, our population was middle aged (median age, 38 years; 20% female), with a mildly elevated BMI (median BMI, 26 kg/m^2^). Of the 97 participants in our study, 49 participants completed all 7 dietary chambers, and 28 completed 6 chambers (Supplemental Figure 1). High carbohydrate overfeeding induced greatest increase in 24-hour EE. Substrate oxidation rate changes were generally consistent with the availability of macronutrients (Figure 2); for example, we observed a shift toward fat oxidation (in lieu of carbohydrate) as quantified by lipid oxidation (LIPOX) rates and a fall in 24-hour RQ in fasting and high-fat diets (with the converse in diets with greater carbohydrate content).

### Shifts in the circulating metabolome reflect molecular pathways of substrate oxidation during different nutritional exposures.

**Figure 3:**
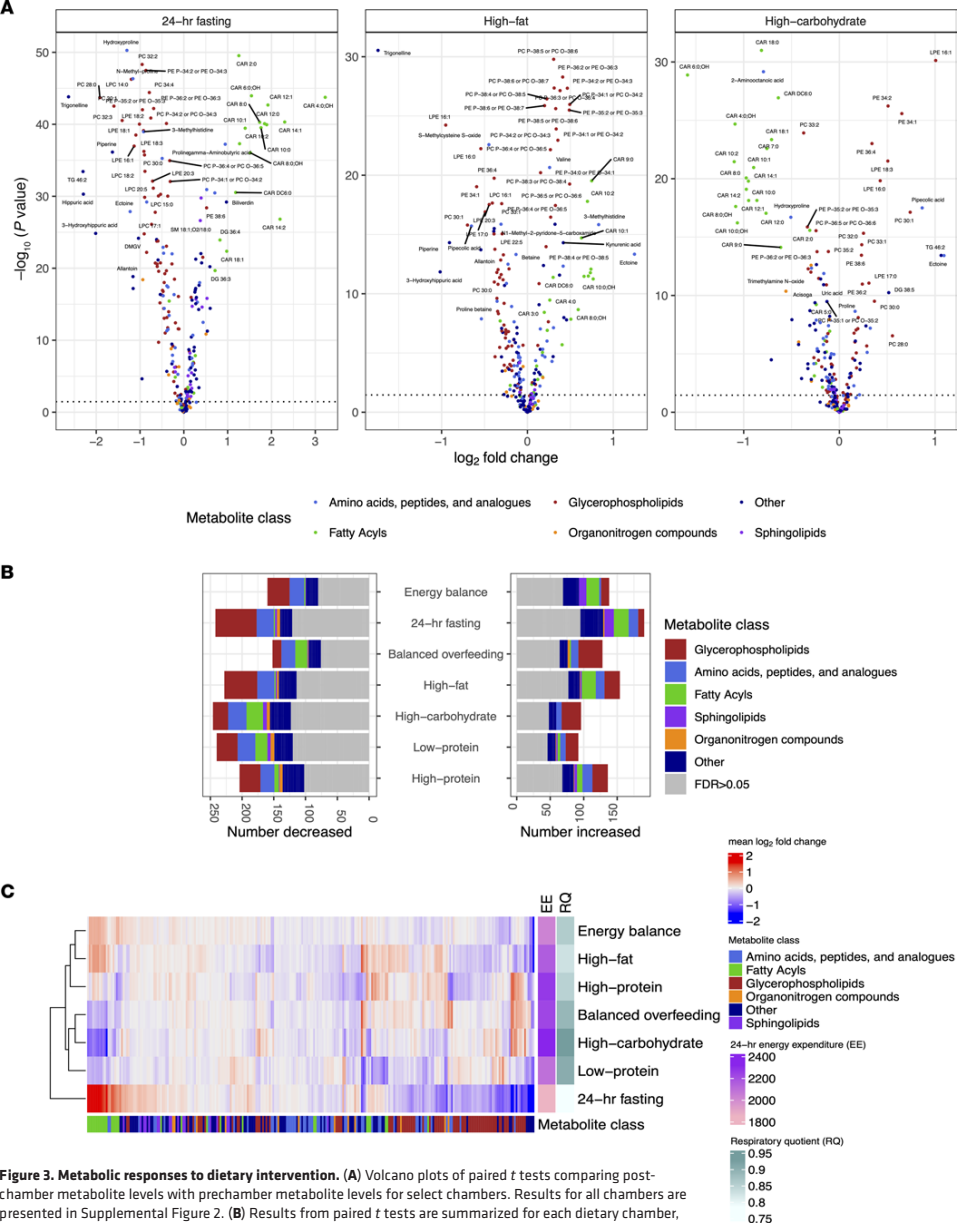
Metabolic responses to dietary intervention. (**A**) Volcano plots of paired *t* tests comparing postchamber metabolite levels with prechamber metabolite levels for select chambers. Results for all chambers are presented in Supplemental Figure 2. (**B**) Results from paired *t* tests are summarized for each dietary chamber, grouped by HMDB metabolite class demonstrating glycerophospholipids as the most common class of metabolite to change. (**C**) Heatmap of the mean log_2_ fold change for all metabolites for all dietary chambers, demonstrating similarities between clusters of diets.

We identified substantial shifts in the assayed circulating metabolome across different 24-hour chambers, broadly consistent with putative effects of macronutrient composition in each dietary prescription (Figure 3, A and B; full results in Supplemental Figure 2 and Supplemental Table 3). The 24-hour fasting condition elicited the most distinct metabolic pattern consistent with known physiology of early starvation, including (a) increases in mitochondrial β-oxidation (increased even medium to long-chain acylcarnitines, pantothenic acid), (b) increased ketogenesis (e.g., C4:0-OH carnitine, a ketone body; ref. 9), (c) increased fatty acid availability (e.g., derived from phospholipid metabolism [phosphatidylcholine/phosphatidylethanolamines (PC/PEs)] to diacylglycerols and sphingomyelins; refs. 19, 20), and (d) generalized decrease in multiple anapleurotic amino acid substrates for TCA cycle flux (e.g., alanine, glycine, threonine, tryptophan, proline, tyrosine) (21). Fasting was associated with increased β-oxidation, demonstrated by the largest increase in C2:0 carnitine (a product of acetyl-CoA; ref. 22) and C4:0-OH carnitine (a ketone body present during acetyl-CoA excess; ref. 23) of any prescribed diet. In addition, we observed a global decrease — not an increase — in lysophosphatidylcholines (LPCs) and lysophosphatidylethanolamines (LPEs) during the 24-hour fast (another byproduct of PC catabolism), consistent with relations of increased LPCs to decreased fatty acid oxidation (24) and broad proinflammatory phenotypes (25). Finally, we observed a decrease in many glucogenic amino acids, consistent with increased liver gluconeogenesis (26) or decreased intake, except for branched-chain amino acids (BCAAs; leucine, isoleucine, valine). The increases in circulating BCAA are consistent with inhibition of their breakdown during increased fatty acid oxidation (via NADH availability) or potentially decreased availability of reaction substrates for BCAA catabolism provided through glycolytic flux (26). Indeed, the lower C3:0 carnitines during fasting — a molecule that is liberated during BCAA catabolism (22, 27) — supports decreased BCAA breakdown in favor of lipid metabolism.

Across all dietary chambers, we observed a generally diverse pattern of metabolite changes, most prominently in amino acids, glycerophospholipids, and fatty acyls (Figure 3B). Of note, there was consistency in dietary metabolite responses by 24-hour RQ that reflected substrate preference during a chamber (Figure 3C); diets associated with a generally lower 24-hour RQ (consistent with increased fat oxidation) exhibited increases in fatty acids (specifically acylcarnitine species) and decreases in glycerophospholipids (predominantly PCs and their precursor PEs or catabolic byproducts LPCs/LPEs; ref. 28). Patterns of metabolite excursions (not necessarily overall fold change magnitude) were similar across diets with similar macronutrient composition, energy intake, or substrate oxidation preferences; metabolic patterns during fasting were correlated directly to energy balance (a lower caloric intake than other chambers, ρ = 0.67) and high fat (similar preference for LIPOX, ρ = 0.34). On the other hand, the relation in metabolic patterns between fasting and diets with greater carbohydrate availability were inverse (high-carbohydrate, ρ = –0.29; balanced overfeeding, ρ = –0.43; Supplemental Figure 3, A and B; full correlation plots in Supplemental Figure 4). High-protein and high-fat diets exhibited concordance, likely owing to a similar fat macronutrient composition (20% versus 30%, respectively). Given the random order, crossover design of chambers after energy balance across the study, the consistent mapping of metabolic responses to physiologic adaptation phenotypes, and consistent results in mixed modeling results (accounting for carry-over design, Supplemental Figure 5 and Supplemental Table 4) increased our confidence in absence of a bias by carry-forward effects of metabolites across chambers. Of note, the low protein chamber exhibited the least concordance in pattern across chambers (Figure 3C). Metabolic changes within the low protein chamber included a generalized decrease in most nonessential amino acids (Supplemental Figure 6), except alanine, glutamine, and glycine, some of which was consistent with free-living studies of a low-protein diet (29). In addition to metabolites that denote substrate utilization, we observed dynamicity in several metabolites of more general clinical interest. For example, dimethylguanidinovaleric acid [DMGV] (30–32) — implicated in hepatic steatosis and metabolic phenotypes in humans — was increased during high-carbohydrate feeding and decreased on fasting, consistent with observational studies linking DMGV levels to sugary beverage consumption (31). Moreover, several specific microbial products (e.g., trimethylamine N-oxide, indole derivatives, hippuric acid; refs. 33–37) potentially implicated in metabolic disease states were dynamic across diets, highlighting unique interconnections among macronutrient intake, host commensal processing, and metabolic response, seen with the metabolome in other studies (1).

### Linking metabolic excursions during nutrition to physiologic responses quantified in human metabolic chambers.

**Figure 4:**
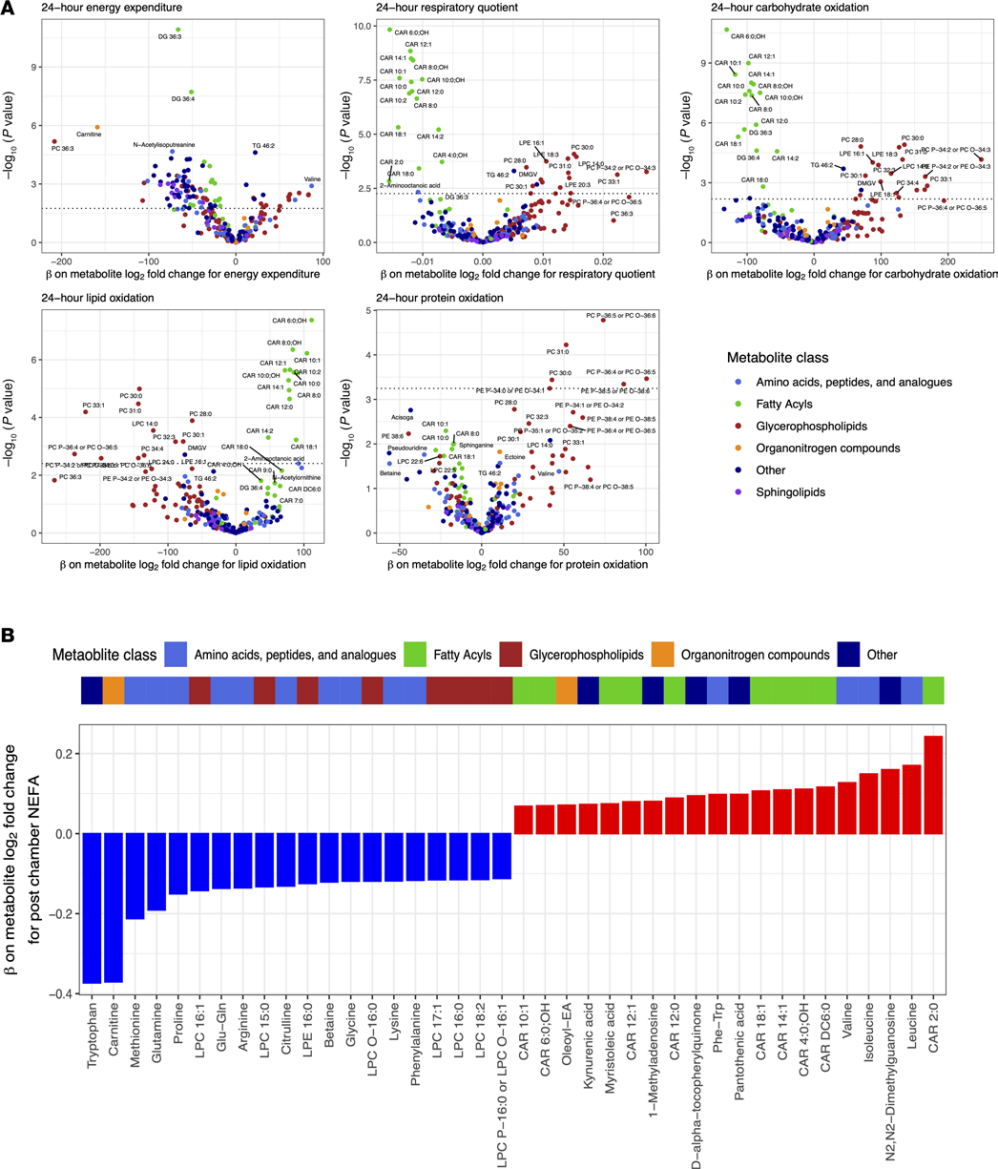
Coordinated metabolomic and physiologic responses to dietary perturbation. Using a linear mixed model for the outcomes of metabolic parameters (e.g., 24-hour energy expenditure, 24-hour respiratory quotient, etc.), we estimated the effect of log_2_ fold changes in individual metabolites aggregated across diets. Example mixed model: 24-hour energy expenditure = log_2_ fold change metabolite + prechamber log_2_metabolite level + diet + age + sex + race + BMI + random intercept per participant. (**A**) Volcano plots of the β coefficients from the log_2_ fold change metabolite variables on respective metabolic parameters. (**B**) Waterfall plot of the β coefficients on metabolite log_2_ fold change for the outcome of postchamber nonesterified fatty acids (NEFA). Model: postchamber NEFA = log_2_ fold change metabolite + prechamber log_2_ metabolite level + prechamber NEFA + diet + age + sex + race + BMI + random intercept per participant.

**Figure 5:**
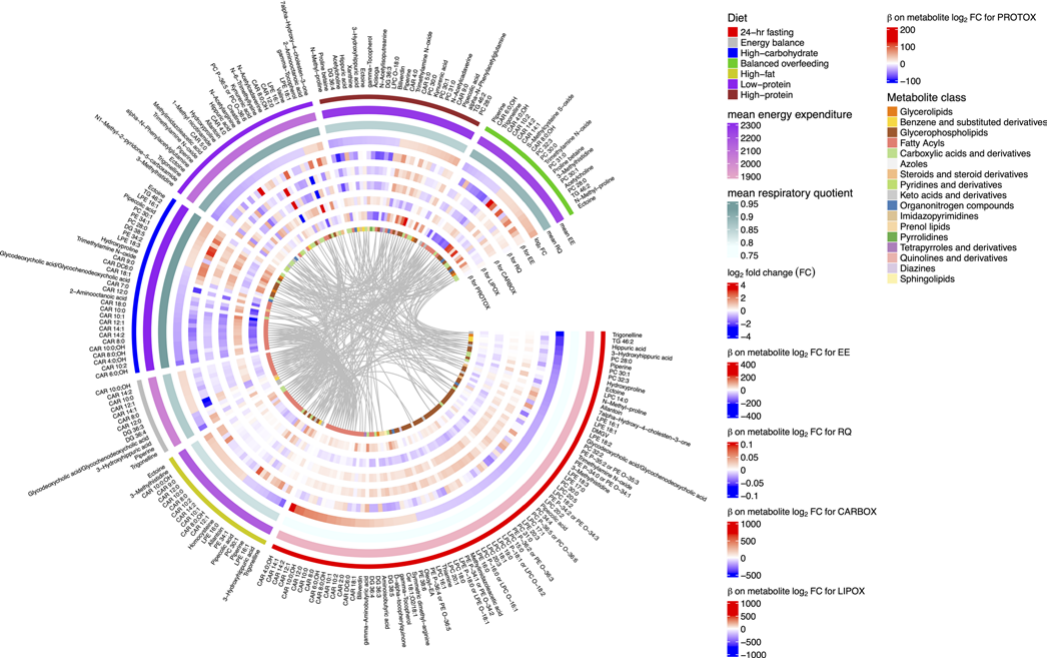
Summary visualization of dietary chamber–related changes in metabolites and relations with changes in global measures of metabolism. For visualization, we present metabolites that with an absolute (mean log_2_ fold change) > 0.5 in any diet with an FDR < 5%. EE, 24-hour energy expenditure (kcal/day); RQ, 24-hour respiratory quotient; FC, fold change; CARBOX, 24-hour carbohydrate oxidation (kcal/day); LIPOX, 24-hour lipid oxidation (kcal/day); PROTOX, 24-hour protein oxidation (kcal/day).

Given these results indicating that metabolomic changes after exposure to 24 hours of dietary perturbations mirror physiologic substrate oxidation preferences (by 24-hour RQ), we explored this directly across diets (Supplemental Table 5). Using mixed models across all participants and chambers to maximize power, we identified metabolites associated with 24-hour RQ, individual measures of substrate oxidation rates, and 24-hour EE (Figure 4A). This analysis was predicated on evidence that — although fuel preference is driven primarily by dietary macronutrient content — there remains an intrinsic intraindividual (within an individual) fuel preference across diets (13, 38) that can only be assessed in these types of controlled cross-over studies. Across the metabolome, prediet chamber substrate availability for lipid mobilization (e.g., acylcarnitines and glycerophospholipids) was associated with substrate preference during the chamber; higher prechamber acylcarnitine and PC concentrations were associated with LIPOX rates and 24-hour RQ, consistent with acylcarnitines as indicators of increased β-oxidation and with a role for PCs as a fatty acid source (39). In addition, metabolic excursions during diet were also closely linked to substrate handling. For example, an increase in several even-chain acylcarnitines was associated with greater 24-hour LIPOX rate (and lower 24-hour carbohydrate oxidation [CARBOX]), consistent with these species as markers of incomplete β-oxidation during mitochondrial fat overload in rodents (22). In addition, both higher prechamber and in-chamber change in C2:0 carnitine were associated with a lower 24-hour RQ, consistent with the greater lipid turnover during fasting, attendant higher mitochondrial acetyl-CoA levels, and subsequent “buffering” of these acetyl-CoAs by conversion to C2:0 carnitine (40, 41). Results were largely robust to regression within each diet separately (Supplemental Figures 7 and 8, and Supplemental Tables 5 and 6). In addition, the relation between prechamber metabolite level and 24-hour RQ and the metabolite change during a chamber with the 24-hour RQ were concordant (ρ = 0.86; Supplemental Figure 9), specifically for metabolites reflecting fatty acid oxidation (acylcarnitines, pantothenic acid) and lipid availability (PC, LPC/LPE). In examining nonesterified free fatty acid (NEFA) levels before and after each chamber as an index of adipose tissue lipid mobilization, we observed a consistent correlation of increased NEFA levels during a chamber with an increase in circulating acylcarnitines, specifically in chambers of caloric excess (Figure 4B, Supplemental Figure 10, and Supplemental Tables 7 and 8). While our study did not serially sample the metabolome during chambers, these findings do provide support for a balance between increased lipid availability (NEFAs) and its utilization (acylcarnitine metabolism) and liberation (PE and PC metabolism). Additionally, we observed a positive association between increase in D-α-tocopherylquinone and increased NEFA, consistent with its role as a vitamin E catabolite that serves as a carnitine-dependent cofactor for mitochondrial fatty acid desaturases (42). The relation of NEFA to changes in other species (including amino acids) was complicated and less uniform across diets, likely owing to macronutrient context-specific interactions between intake and metabolism. Unlike 24-hour RQ, 24-hour EE did not map as consistently to the changes in the assayed metabolome with diet (Figure 4A). Figure 5 represents a full summary of our results, highlighting these shared relations in physiologic-metabolic phenotype across diets.

## Discussion

Substantial literature charting metabolic substrate flux over 6 decades has delineated a roadmap for cellular metabolism through detailed perturbational studies in mammalian systems (43). Most studies in humans that link substrate metabolism to organism-wide physiology, however, are more limited, challenged by the method used to measure these physiologic phenotypes with the required high precision and accuracy while controlling confounding and nutritional exposure. In this context, the application of metabolite profiling has emerged as a quantitative “snapshot” of human metabolism that can be related to dietary patterns to begin to clarify responses to nutrition (44–47). The primary role for metabolite profiling in this space has been within large epidemiologic studies, providing large sample sizes (required to power associations with 1 × 10^2^ to 1 × 10^3^ metabolites) with less control over nutritional exposure and characterization and no gold-standard methodologies to quantify substrate metabolism.

The goal of our study was to provide a set of reference data from precise, high-quality human metabolic chambers to delineate the relation of a circulating metabolome and its changes to targeted nutritional exposures. Our primary result establishes functional interconnections between metabolic and physiologic responses in humans. We observed broad shifts in the metabolome across diets, largely linked to substrate availability and processing and with a striking consistency across participants. Results from the 24-hour fasting condition extended prior metabolomic findings (9), linking mitochondrial β-oxidation and fatty acid availability directly to 24-hour LIPOX rates in humans, defined by gas exchange and urinary measures. Our 24-hour diet interventions yielded consistent physiologic and metabolic alterations with regard to lipid metabolism. Specifically, diets associated with a lower 24-hour RQ (greater fat oxidation) were associated with increases in β-oxidation flux (acylcarnitines) and adipose tissue fatty acid mobilization (NEFAs) and with decreases in glycerophospholipids (a substrate source), consistent with coordinate substrate management during enhanced β-oxidation. Conversely, metabolic shifts during fasting or fat overfeeding relative to diets with greater carbohydrate availability were inverse. Collectively, the current report provides a resource for metabolite changes during differing macronutrient diets, highlighting the importance of fat metabolism. These results extend decades of metabolic research linking molecular metabolic changes and human substrate oxidation in humans in an exquisitely controlled setting, providing important reference data for subsequent metabolic challenge studies.

Metabolomics has been broadly applied to human nutrition to establish biomarkers of diet (48–50) and to assess the effect of different diets on human metabolism (9, 51–53). In this context, prospective interventional studies are thought to offer more close control over nutritional exposure and confounding, though adherence and economic limitations remain, limiting sample sizes. Although a full review of the extant literature on metabolomics and dietary studies is out of scope here (reviewed in ref. 54), a key limitation to all controlled studies of feeding is heterogeneity introduced by the effect of individual prescribed foods beyond macronutrients (e.g., a similar macronutrient composition across diets of different foods), the role of exposure duration (e.g., long-term effects of diet on microbiome; ref. 55), and interindividual variability in how foods may be processed (e.g., microbial flora). Despite these limitations, we observed broad consistency in the dynamic metabolome, with changes seen in smaller controlled-feeding studies of single macronutrient exposure. In a cross-over study of 12 healthy volunteers, high-fructose diets (1 week duration) led to a reduction in circulating acylcarnitines and an increase in glycerophospholipid levels (56), similar to our result during a 24-hour high-carbohydrate overfeeding. Similarly, high-fat diet exposure and starvation have been shown in small studies to led to shifts in acylcarnitine metabolism (23) and broad lipid alterations (9), largely consistent with findings here. Nevertheless, outside of starvation studies (where the diet “exposure” is identical), metabolomic shifts are broad (in some studies up to approximately 50% of the assayed metabolome; ref. 51) and dependent on the way a given macronutrient composition is delivered (57), highlighting the complexity of nutrition research and difficulty in discerning clear physiologic interpretations.

The current study addresses these limitations by (a) directly measuring the effect of a variable macronutrient composition on substrate metabolism and EE under (b) exquisitely controlled conditions while participants resided in a domiciled unit (e.g., weight, sleep, activity, temperature) across (c) multiple dietary interventions in a cross-over framework to optimize power with (d) gold-standard indirect calorimetry. The sample size here is large for a human chamber-based study, given its participant time intensive nature (nearly 40-day inpatient stay) and cost. While we prioritized standardization of macronutrients (not specific foods), the precision of 24-hour chamber measures of substrate oxidation alongside metabolites along canonical pathways of substrate metabolism allowed us to link substrate availability/processing to physiology. We observed opposite metabolic and physiologic changes (24-hour RQ, 24-hour substrate oxidation profiles) during high-carbohydrate relative to high-fat/fasting diets that directly map to metabolism. As an example, we found a rise in even-chain acylcarnitine species during high-fat overfeeding and fasting, and concentrations of several even-chain acylcarnitines were associated with chamber-measured LIPOX rates and postchamber NEFA levels, consistent with incomplete β-oxidation during mitochondrial fat overload as noted in rodents (22). Interestingly, a higher prechamber and chamber-related change in C2:0 carnitine was associated with lower 24-hour RQ, and higher levels of C2:0 carnitine were observed in the fasting chamber (intense LIPOX), in support of its role as a “buffer” for mitochondrial acetyl-CoA. Indeed, circulating concentrations of C2:0 carnitine (the “parent” acylcarnitine synthesized from L-carnitine) mirror its intracellular profile, thought to serve as a pool for “storing” increased mitochondrial acetyl-CoA during conditions of high-lipid metabolism (e.g., fasting) (40, 41). Conversely, C2:0 carnitine decreased during the high-carbohydrate chamber, consistent with decreased mitochondrial acetyl-CoA (less ingested fat substrate) and requirement for removal of acetyl-CoA–mediated inhibition of pyruvate dehydrogenase, thereby allowing substrate “flexibility” from fat to glycolytic metabolism (41). Moreover, changes in BCAA metabolism and downstream acylcarnitines were in concert with changes in markers of lipid metabolism, suggesting an orchestrated response to substrate availability within and across individuals. While several of these results are supported by a wealth of model system and human studies, the current prolonged, inpatient human metabolic chamber approach provides an exquisitely controlled setting to underscore links between substrate metabolism (at the mitochondrial level) and alterations in representative circulating metabolites.

The results of this study ought to be viewed in the context of its design. First, our protocol prespecified macronutrient content, allowing for some titratable food prescription to optimize participant tolerance, an approach that is not uncommon in studies of macronutrient responses but does limit inference on food-related metabolites. Despite exquisite control over confounding and allowance of washout periods between diets, randomization of chambers in a crossover design may still exhibit carry-forward effects (wherein one chamber’s results affect a subsequent one), though our results were consistent even when accounting for chamber order in mixed models. Finally, while our metabolite profiling platform was broad, it excluded some metabolites that may have been of physiologic interest (e.g., TCA cycle intermediates, fatty acids). Nevertheless, the consistency of signals with fat oxidation and 24-hour RQ across diets and evidence of replication in other studies are encouraging (9, 23, 56). These limitations highlight the inherent complexities in studying human nutrition at precision scale, where large studies of prolonged, controlled feeding are logistically, ethically, and financially impractical.

An important unanswered question from these studies revolves around understanding whether interindividual variability in metabolic responses can be tied to future weight gain. The unique power of this study — precision of repeated physiologic and metabolic measures during controlled dietary conditions — fundamentally limits sample size (and the feasibility of larger-scale studies). While the crossover study design and repeated measures allowed an increased power to detect average effects across participants and diets, assessment of diet-specific responses (and how factors like obesity, age, sex, and other social-demographic indices affect these responses) remains underpowered. Similar constraints exist in applying chamber data to understand metabolomic responses’ effect on weight gain, a concept that has required much larger sample sizes (58), or studying extremes of physiologic responses to diet. In this regard, upcoming results of large, targeted metabolomic validation efforts of nutrition at population scale (e.g., Nutrition for Precision Health initiative) will be sufficiently powered with social-demographic diversity required to provide rigorous population-level estimates of dietary effects that inform individual responses to diet and implication on metabolic risk. The results presented here from human metabolic chambers provide important relief and context for these ongoing results and detailed, controlled extension of prior results over decades of studies as a starting point for integrated, hypothesis-driven studies of human metabolism.

## Methods

### Sex as a biological variable.

Our study enrolled males and females. We adjusted for sex in models to account for sex as a biological variable. Of note, our study design necessitated a longitudinal modeling approach, wherein each patient is their own control (allowing for intraindividual control).

### Study cohort.

This study involved a NIH-approved protocol to study short-term metabolic adaptation under macronutrient stress (NIH protocol no. 07-DK-N215) (13, 18, 59, 60). Of note, 94 participants had valid data for energy balance respiratory chamber data used to calibrate the 200% caloric excess chambers. Subsequent 24-hour intervention diets were randomized. Due to technical issues or participant withdrawal, the number of participants in each dietary chamber varies (Table 1). Participants were required to have a stable weight for 6 months and be otherwise healthy based on medical history and physical examination upon admission to the inpatient NIH Clinical Research Center (Supplemental Figure 11). Upon admission, volunteers were placed on a WMD, which was followed prior to and in between the 24-hour intervention diets (13). Participants were weighed daily, and calories were adjusted to maintain weight (coefficient of variation of weight over study 0.9% ± 0.6%). After 3 days of the WMD, participants underwent a 75-gram oral glucose-tolerance test to exclude individuals with type 2 diabetes (T2D) or impaired glucose regulation (fasting glucose ≥ 100 mg/dL or 2-hour glucose ≥ 140 mg/dL; T2D, fasting glucose ≥ 126 mg/dL or 2-hour glucose ≥ 200 mg/dL). The participants then underwent a series of 24-hour whole-room indirect calorimetry experiments (chambers) to quantify 24-hour EE and 24-hour RQ. A 24-hour urine collection was performed to assess protein oxidation rates, and lipid and CARBOX were calculated as described (61). Chambers were performed under several different conditions, including energy balance (calories ingested = calories expended), fasting, and 200% caloric overfeeding diets in random order and with intervening periods of at least 3 days on WMD: (a) standard overfeeding (SOF); (b) a low-protein overfeeding (LPF); (c) high-fat/normal-protein overfeeding (FNP); (d) high-fat/high-protein overfeeding (HPF); and (e) high-carbohydrate/normal-protein overfeeding diet (CNP). Caloric excess (200% of individual-specific energy needs) was used to perturb energy balance and to best elicit interindividual heterogeneity in phenotypes. The total inpatient stay lasted approximately 37 days.

### Dietary exposures.

Macronutrient composition for each diet is shown in Supplemental Table 1. Carbohydrates were a mixture of simple (e.g., soda, candy) and complex (legumes, vegetables, and fruit). Protein source was predominantly of animal origin. Dietary exposures included 24-hour fasting and different overfeeding conditions (200% of calories expended during EB), and fasting diet macronutrient composition was quantified by “The Food Processor” software (ESHA Research). Residual uneaten food was returned to the metabolic kitchen to calculate actual macronutrient intake during each session. These dietary compositions were chosen specifically to stress the metabolic system not only by providing modern obesogenic diets (e.g., high-carbohydrate or high-fat) but also based on previous data indicating that low-protein (3%) diets amplify interindividual differences in thermogenesis (62).

### Metabolic chamber measures.

The description of our indirect calorimetry apparatus (63) and methods (13, 18) have been published. The first 2 chamber sessions were used to precisely establish the individual level of energy balance (EB), a condition where isocaloric intake matches EE. Measured 24-hour EE during the first eucaloric chamber was used as the energy intake calories for the second chamber to precisely achieve energy balance. This second chamber was used as the EB chamber in this analysis. The order of subsequent chambers was randomized (to limit confounding effects of the order of dietary exposure on metabolism) and spaced by 3-day intervals of WMD and limited physical activity (walking, playing pool, watching TV) to limit “carry-forward” effects from prior a chamber’s diet. Chambers sessions in which participants did not consume > 95% of the food provided by the metabolic kitchen were withdrawn from the analysis.

Participants entered the chamber immediately following breakfast at 7:00 a.m. (if not a fasting chamber day). Venous blood was drawn before entry into chamber and upon exit into EDTA tubes, with DPP-IV inhibitor and aprotinin, centrifuged for 10 minutes at 4°C at 1,436*g*–1,512*g* for plasma generation and stored at −70°C. Meals in the chamber were provided via a 2-way airlock at 11:00 a.m., 4:00 p.m., and 7:00 p.m. Patients were instructed not to be physically active in the calorimeter to limit the contribution of activity to adaptive thermogenesis. Radar was used to monitor physical activity (denoting percentage of time with motion). The temperature of the chamber was controlled at 24°C, and monthly validation tests (involving propane combustion inside the chamber) verified O_2_ and CO_2_ recovery within 2% based on change in propane weight. Air output from the chamber was sampled every minute and compared with inflow (fresh) air to calculate a patient’s CO_2_ production (VCO_2_) and O_2_ consumption (VO_2_) per minute. These measures were utilized to quantify a per-minute RQ (VCO_2_/VO_2_) and the rate of EE (Lusk equation: VO_2_ × 4.686 + [calculated RQ – 0.707] × 0.361/0.293), as described (63, 64). The per-minute values for EE and RQ were extrapolated to 24-hour (multiplied by 1,440). Twenty-four–hour urinary nitrogen excretion rate was measured while in the chamber to estimate 24-hour protein oxidation rate and was used to derive the nonprotein RQ, which was used to calculate 24-hour CARBOX and LIPOX rates (61) as a secondary measure of substrate oxidation preference. We have demonstrated excellent reproducibility of metabolic measures previously (60).

### Metabolite profiling.

Measurement of 321 metabolites, including amino acids, acylcarnitines, and other cationic polar metabolites (Supplemental Table 2), were made using liquid chromatography–tandem mass spectroscopy (LC-MS) as described previously (65). We observed an excellent coefficient of variation in pooled QC samples across metabolites using raw, not log-transformed, data (median 4.7%, 25th–75th percentile, 3.4%–7.6%). We reviewed distributions of the prechamber metabolite levels for each dietary chamber and excluded measurements that were > 5 SDs within a diet. We excluded metabolites with any degree of missingness from our analysis (including those missing due to outlier removal), leaving 263 metabolites for analysis. We log_2_ transformed metabolite levels prior to use in models for model interpretation to be centered around log_2_ fold changes in metabolite levels.

### Statistics.

While our sample size is large for studies of this degree of precision phenotyping in 24-hour metabolic chambers, we were sensitive to dimensionality and overfitting concerns, given the number of metabolites and chambers. In this regard, we performed 2 approaches to test the effect of each dietary chamber on changes in metabolite levels: a serial pre- versus postchamber metabolite comparison (2-tailed *t* test) and a more complex mixed-model approach including interaction terms to model random chamber order and account for cross-over effects. For *t* tests, a FDR of 5% (Benjamini-Hochberg) was imposed across all tested metabolites within a dietary chamber. To estimate the effect of each dietary chamber on the log_2_ fold change in metabolite (in relation to the energy balance chamber), we constructed linear mixed models of the form: postchamber metabolite log_2_ level = prechamber log_2_ metabolite level + diet + chamber order + random effect per participant. Chamber order refers to the order the participant entered the dietary chamber (e.g., energy balance is 1 for all participants, as this was the first chamber entered; the remaining chambers are in random orders across participants). The diet variable was structured with energy balance diet as the referent.

To test the relations of changes in metabolites with physiologic measures including 24-hour EE, 24-hour RQ, and oxidation subtypes, we used linear mixed models. This approach combines data from all dietary chambers into 1 model with repeated measures. As an example, the model for 24-hour EE was of the following form: 24-hour EE = log_2_ fold change metabolite + prechamber metabolite log_2_ level + diet + age + sex + race + BMI + random effect per participant. We compared these models to linear models, stratified by diet of the following form: 24-hour EE = log_2_ fold change metabolite + prechamber log_2_ metabolite level + age + sex + race + BMI, where each model was restricted to data from a single dietary chamber. To test the relations of changes in metabolites with changes in NEFA, we created analogous sets of mixed and linear models, for the outcome of postchamber NEFA with adjustment for prechamber NEFA, prechamber log_2_ metabolite level, age, sex, race, and BMI (the mixed model again had a random effect per participant).

### Study approval.

This study was approved by the IRB of the NIH (Bethesda, MD; NIH protocol no. 07-DK-N215) and Vanderbilt University Medical Center. All participants provided written informed consent.

### Data availability.

The metabolic and phenotypic data from this study are available via controlled access at NIH due to participant confidentiality and inclusion of protected groups. Requests for data should be made to the corresponding author directly, with subsequent approval by NIH. Code utilized in these analyses are available at https://github.com/asperry125/MetFlex (commitID, 31b37139a406c86abcfaa935952d8d2aaf17a9a9). Values for data points in figures are reported in the Supplemental Data Values file.

## Author contributions

RVS, VLM, and JK obtained funding for and supervised the work. ASP, PP, and SH are co–first authors. Order of authorship was decided based on the fact that ASP performed the quality control on metabolite data, coded the statistical models, prepared figures for the manuscript, and wrote the first draft of the manuscript; PP prepared and curated the clinical data and contributed to the manuscript writing; and SH designed the statistical models used. ASP was primarily responsible for statistical analysis, data interpretation, and writing the manuscript; PP was responsible for acquisition and analysis of metabolic chamber data; SH was responsible for designing statistical analysis pipelines, specifically with regard to crossover design methods. MN, JF, KEN, JEB, CBC, VLM, JK, SH, PP, ASP, and RVS contributed to data interpretation and writing and editing the manuscript.

## Supplementary Material

Supplemental data

ICMJE disclosure forms

Supplemental tables 1-8

Supporting data values

## Figures and Tables

**Table 1:**
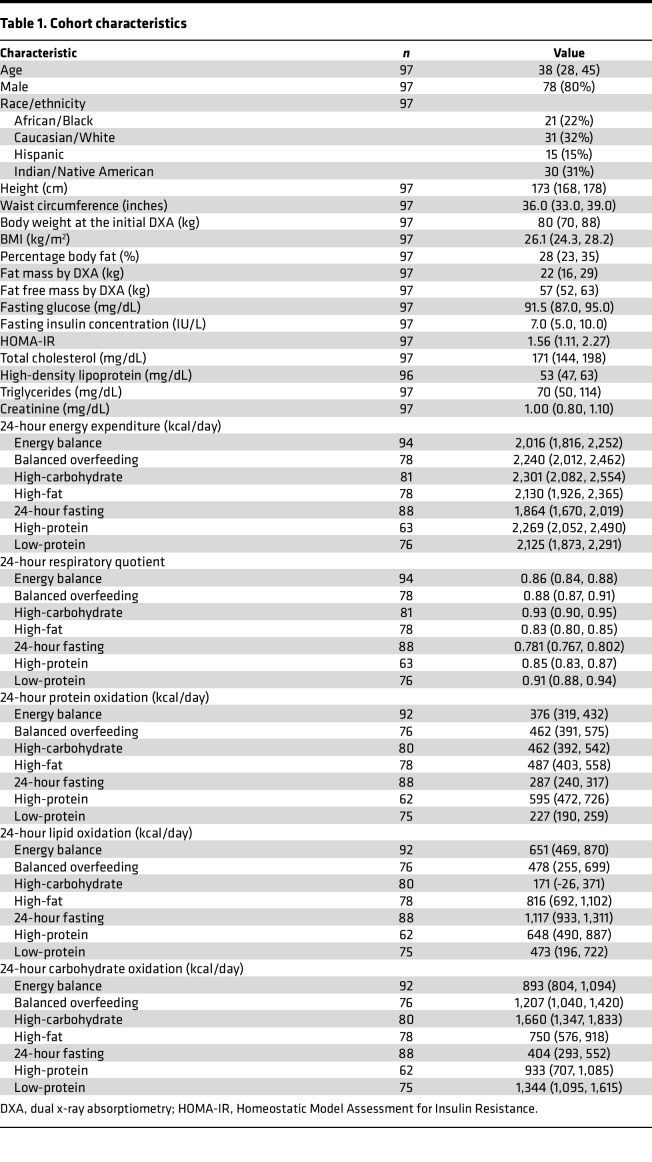
Cohort characteristics

## Version 1 11/22/2024

Electronic publication
