# Supplementary material for: Human metabolic chambers reveal a coordinated metabolic-physiologic response to nutrition
Source: JCI Insight. 2024 Nov 22;9(22):e184279. doi: 10.1172/jci.insight.184279 (PMC11601946; doi:10.1172/jci.insight.184279)
Supplement: Supplemental data [file jciinsight-9-184279-s041.pdf]

Human metabolic chambers reveal a coordinated metabolic-physiologic response to nutrition

Supplemental Material

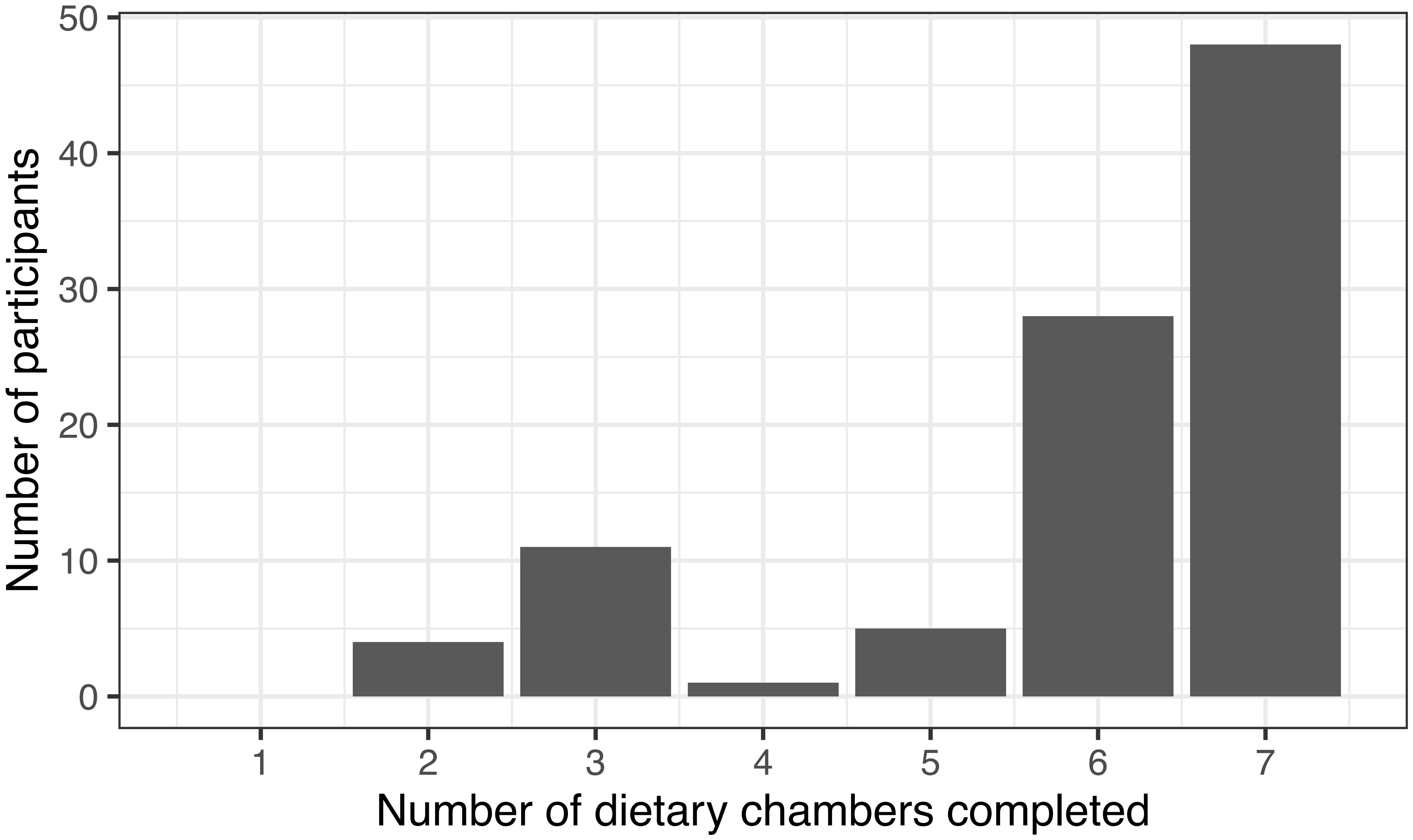

Supplemental Figure 1: Number of dietary chambers completed by study participants.

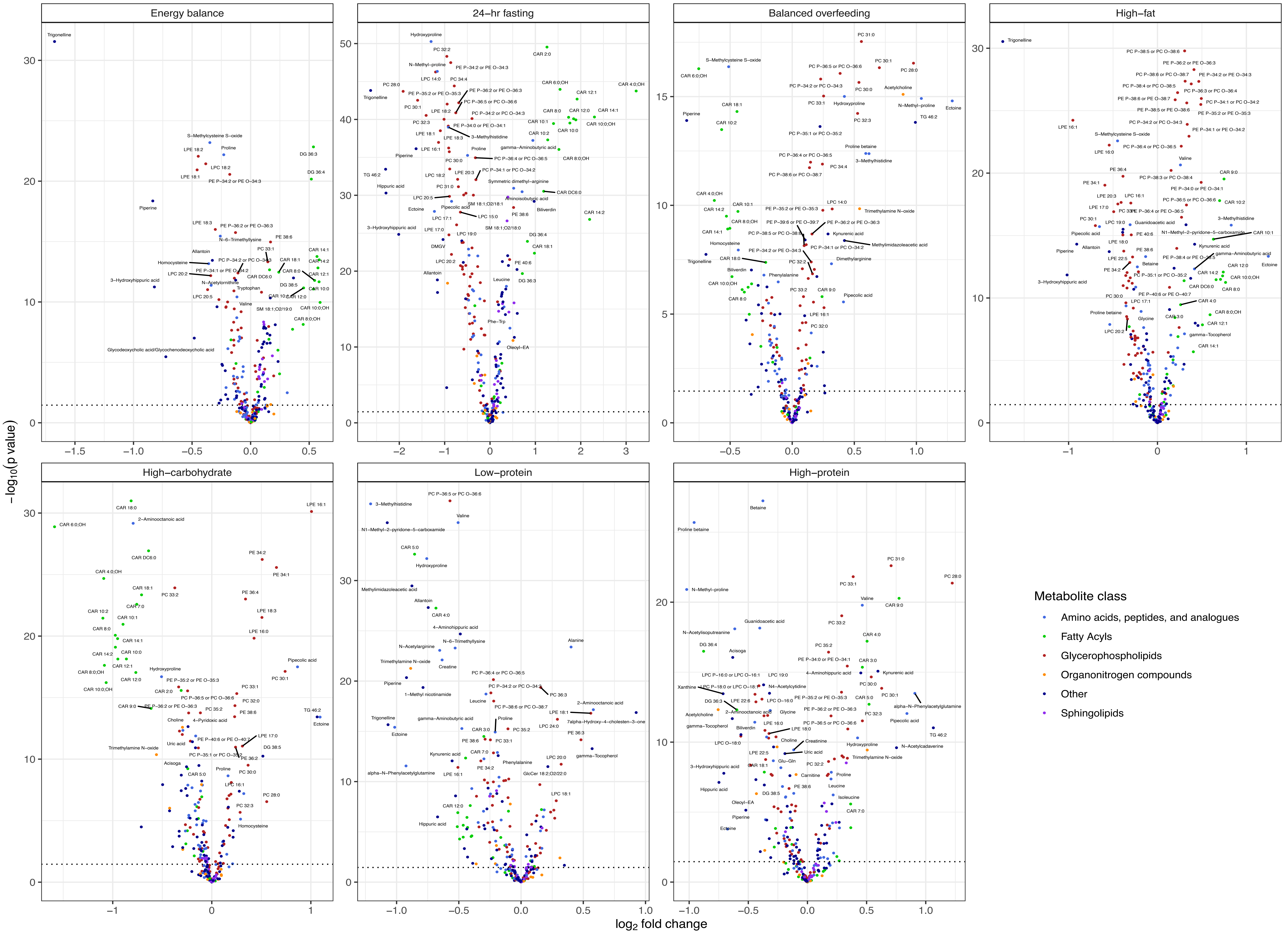

Supplemental Figure 2: Full results from *t*-tests across all 7 dietary chambers. The horizontal dotted line represents a false discovery rate of 5%.

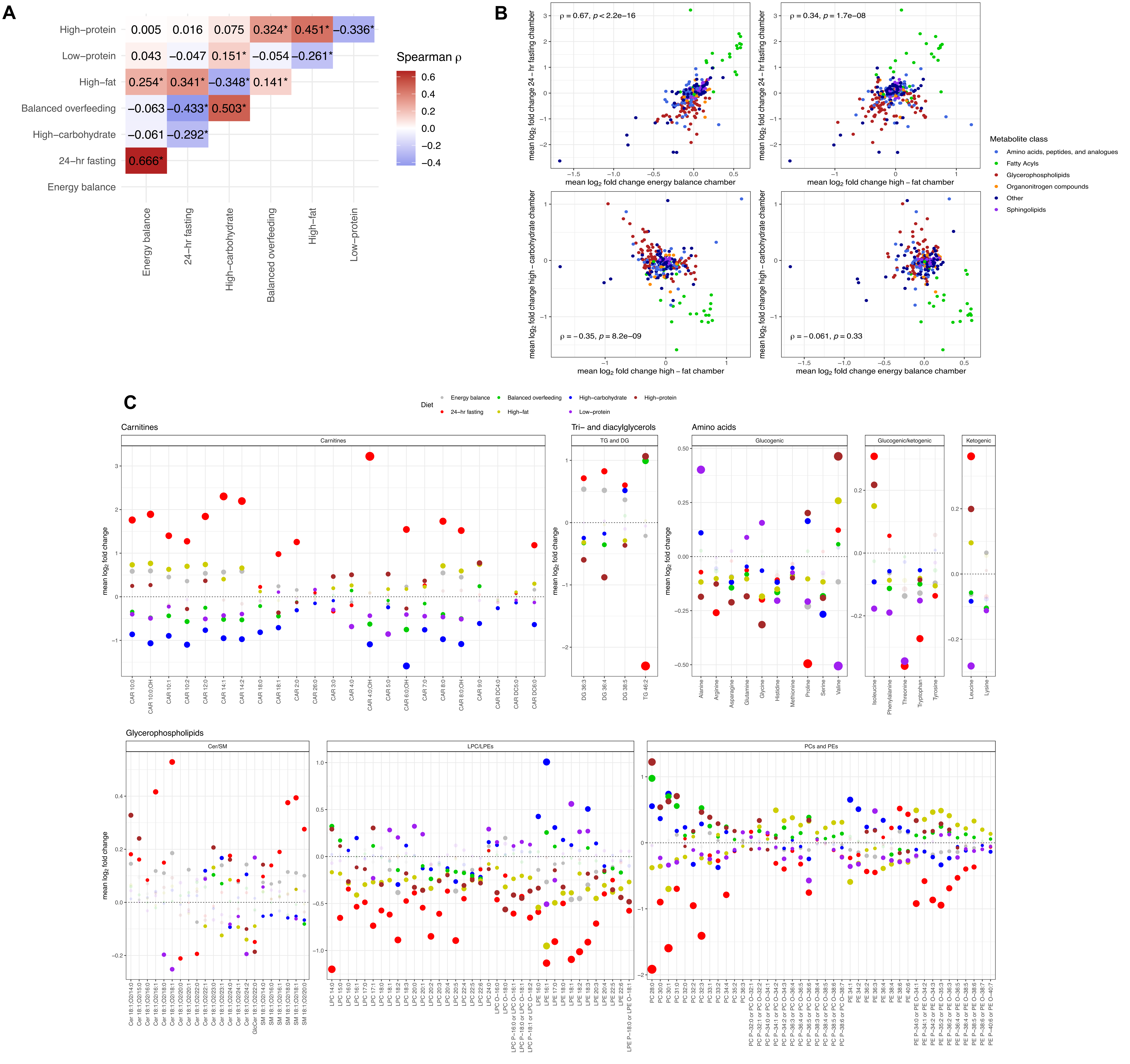

Supplemental Figure 3: Relation of metabolic response across dietary exposure. (A) Spearman correlation heatmap of the paired *t*-test results across dietary chambers. As might be expected, we observed a moderate degree of correlation between the high-carbohydrate and balanced overfeeding diets. **(B)** Select examples from **Panel C** demonstrating correlation of log_2-fold changes between diets. **(C)** Comparison of the mean log_2 fold change of metabolites across all 7 dietary chambers. Colors represent the dietary chamber, size is proportional to mean log_2-fold change, and points are faded if the mean log_2-fold change for each diet-metabolite combination did not reach at an FDR<5%.

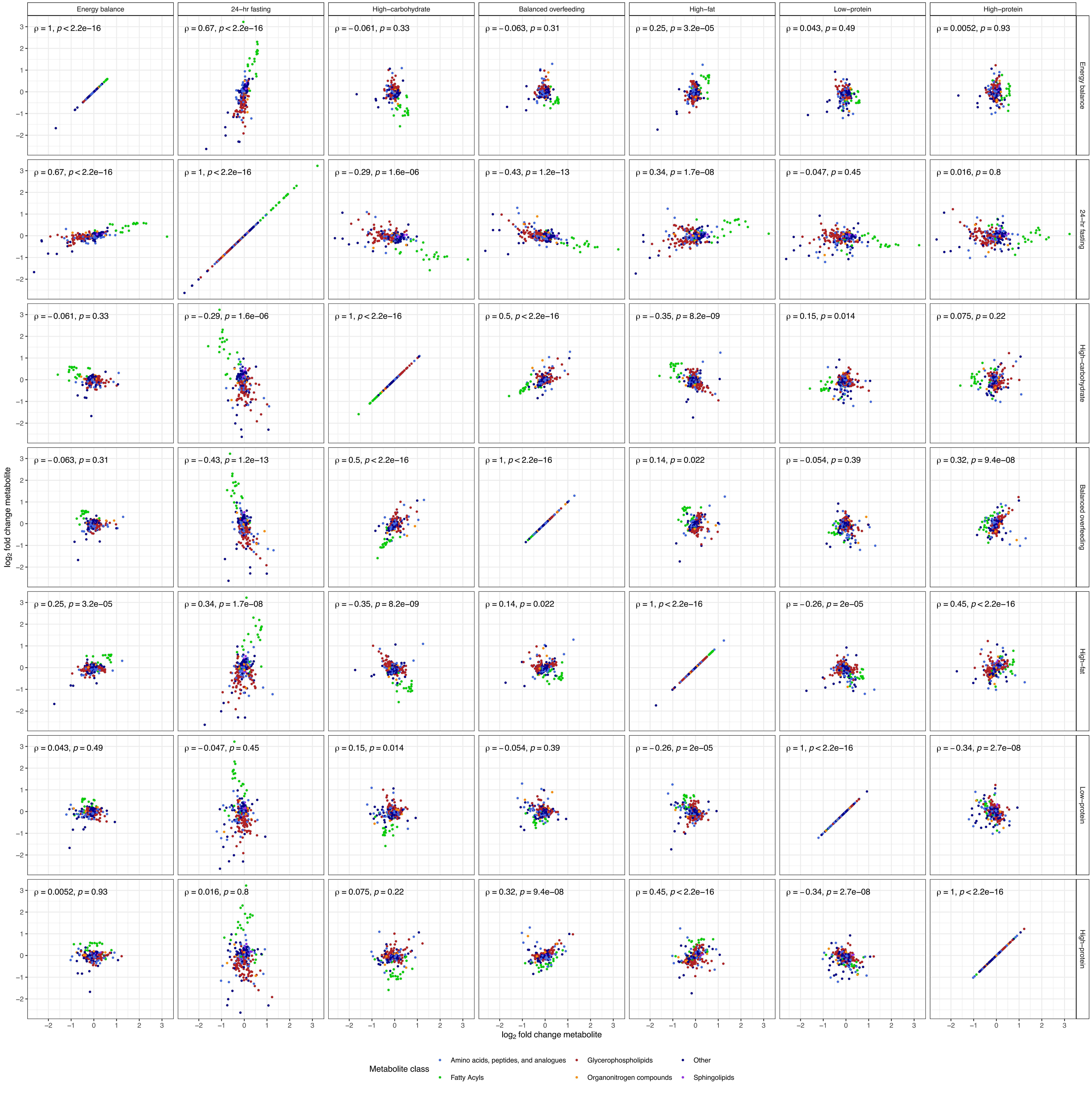

Supplemental Figure 4: Correlation of mean log2 fold changes across all dietary chambers. Spearman correlation plots comparing all diets against each other. This presents the full data which is summarized in Supplemental Figure 3A.

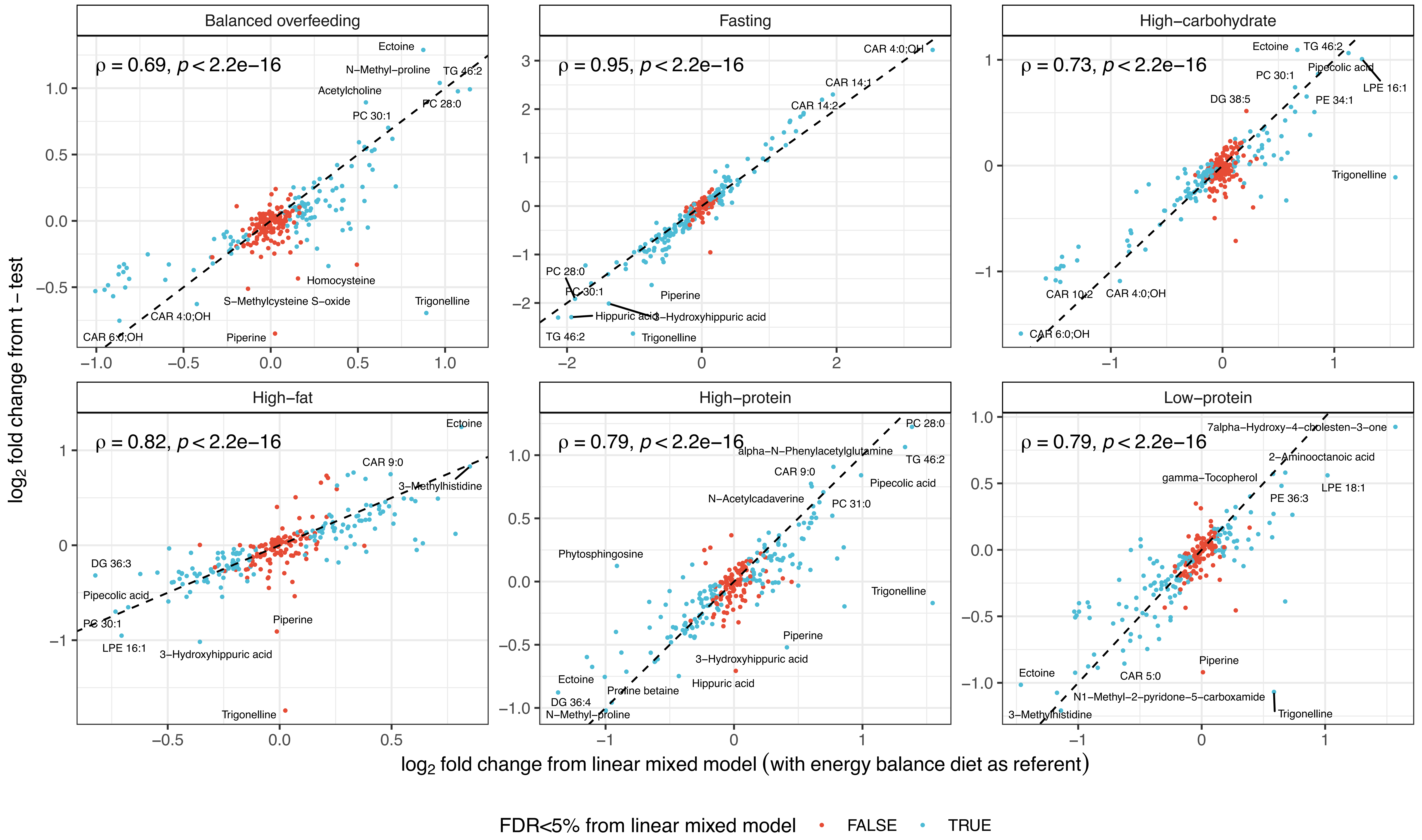

Supplemental Figure 5. Comparison of estimated log2 fold change in metabolite from t-tests and a linear mixed model. Paired t-tests demonstrated numerous changes to circulating metabolites for each dietary chamber (Figure 3A-B). In order to estimate the effect of each dietary chamber on the log2 fold change in metabolite, we constructed linear mixed models as follows: post-chamber log2 metabolite level ~ pre-chamber log2 metabolite level + diet + chamber order + random effect per participant. Chamber order refers to the order the participant entered the dietary chamber (e.g., energy balance is 1 for all participants as this was the first chamber entered, the remaining chambers are in random orders across participants). The diet variable was structured with energy balance diet as the referent. The figure presents the estimated effect of the dietary chamber on the log2 fold change of each metabolite, compared to the energy balance chamber (x-axis). These effects are compared against what was obtained via paired t-tests (y-axis).

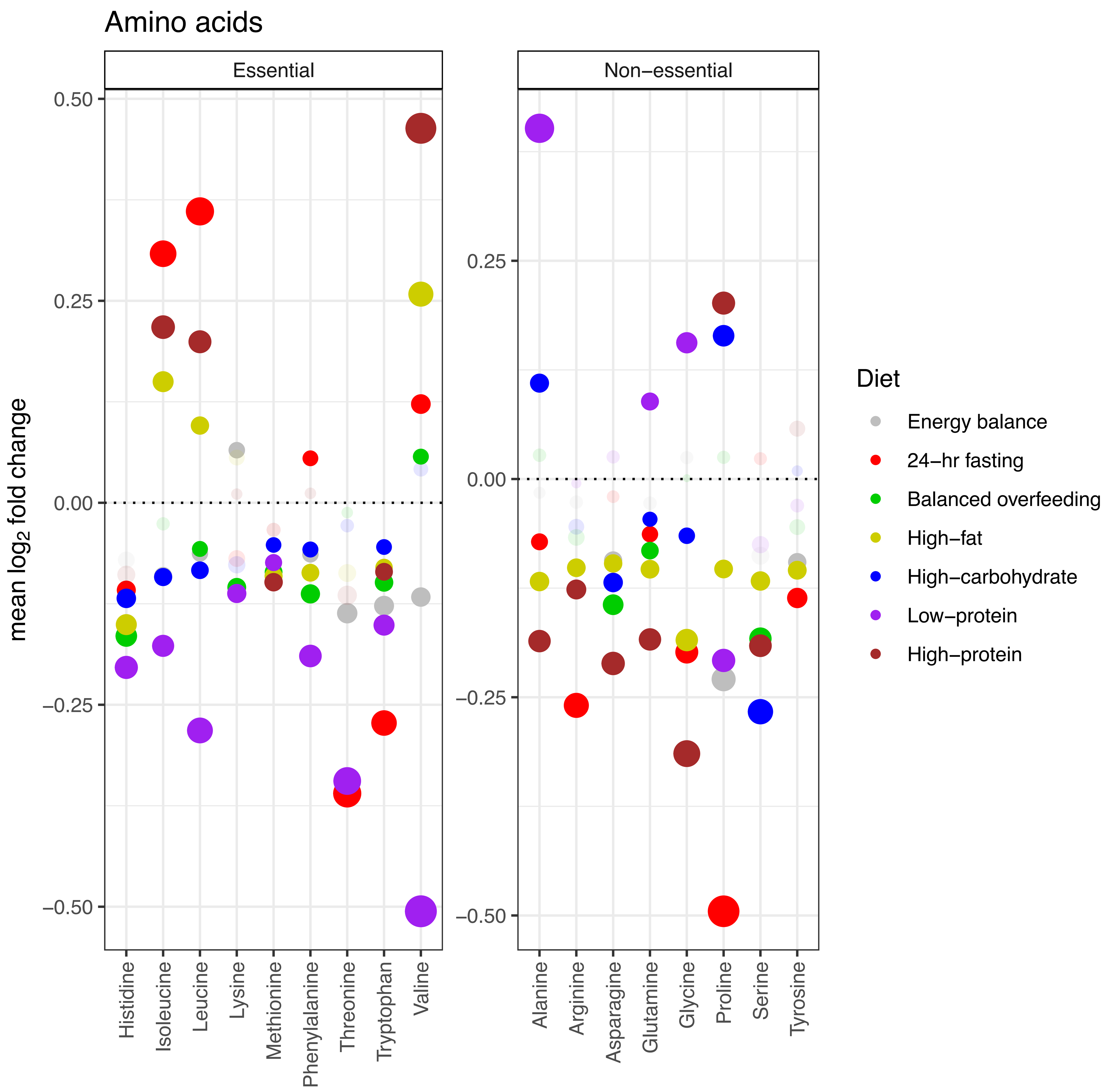

Supplemental Figure 6. Changes in essential v. non-essential amino acids. Comparison of the mean log2 fold change of essential and non-essential amino acids (which were quantified in our study) across all 7 dietary chambers. Colors represent the dietary chamber, size is proportional to mean log2 fold change, and points are faded if the mean log2 fold change for the diet-metabolite combination did not reach an FDR<5%.

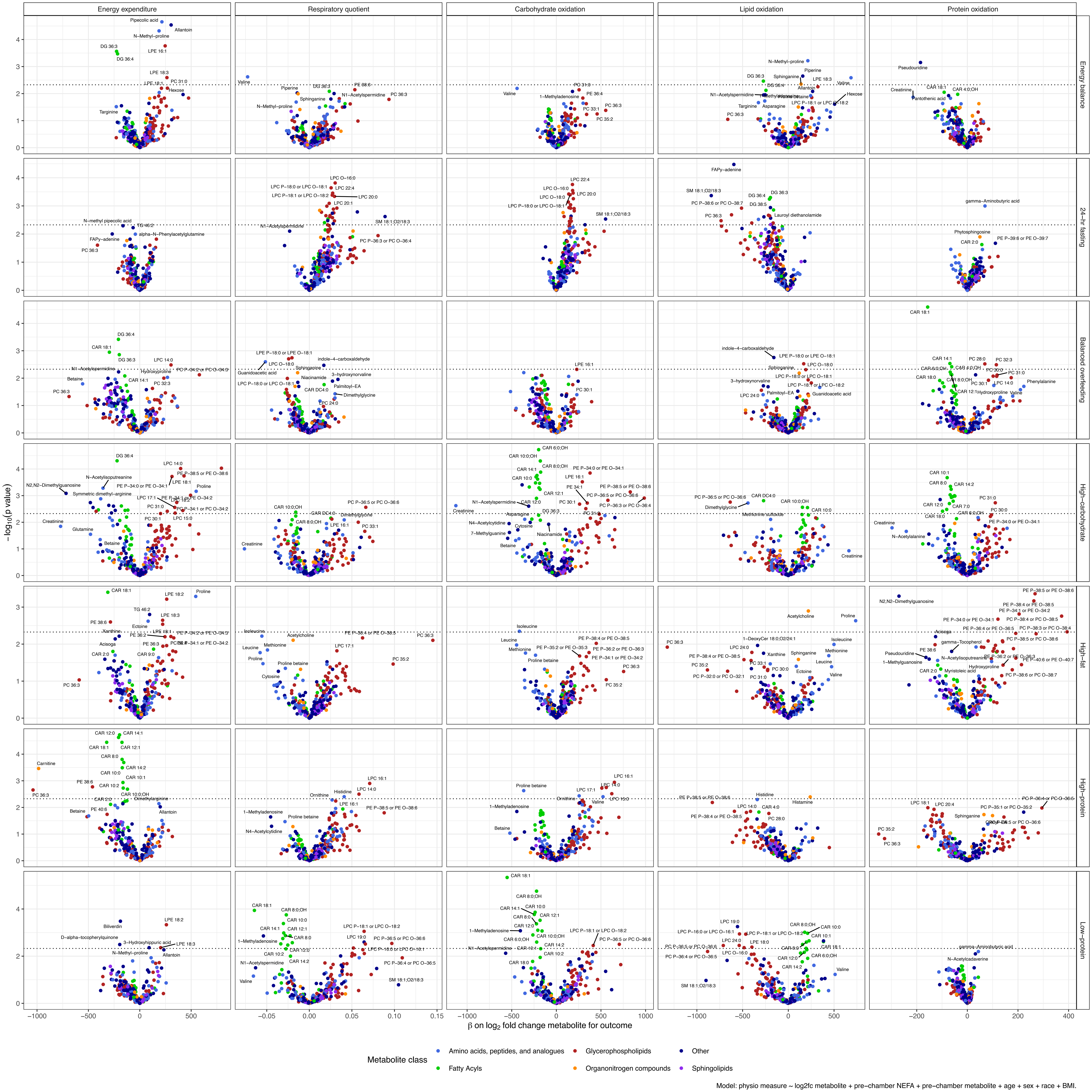

Supplemental Figure 7: Relationship between $\log_2$ fold change metabolite and physiologic measures by diet. Volcano plots reporting the beta coefficient on $\log_2$ fold change metabolite as a predictor of physiologic measures, such as 24-h energy expenditure and 24-h respiratory quotient. Example model: 24-h energy expenditure ~ $\log_2$ fold change metabolite + pre-chamber $\log_2$ metabolite level + age + sex + race + BMI.

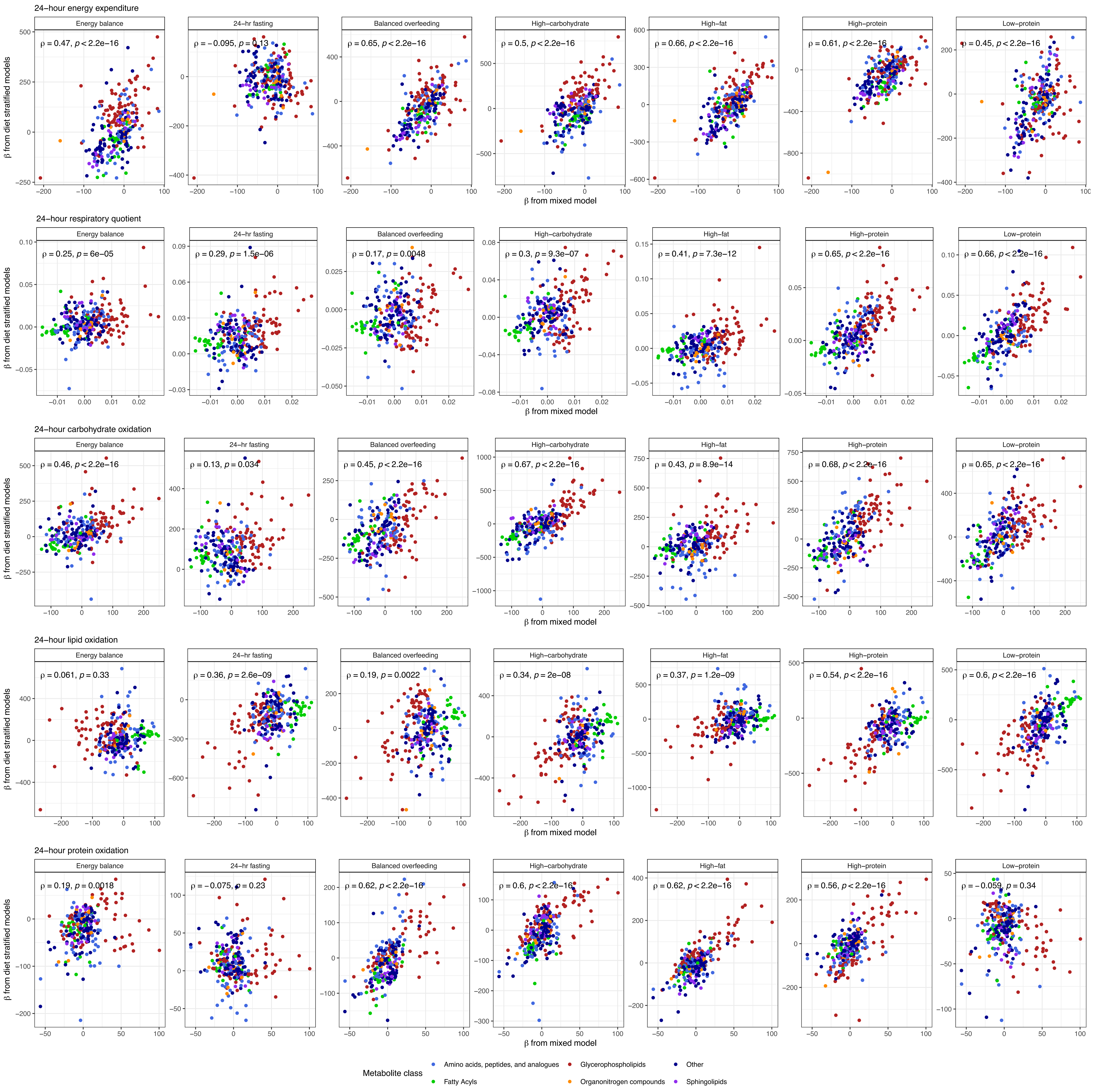

Supplemental Figure 8: Comparison of mixed model with diet stratified linear models. Beta coefficients on the x-axes come from linear mixed models of the structure: outcome (e.g., energy expenditure) ~ $\log_2$ fold change metabolite + pre-chamber $\log_2$ metabolite level + diet + age + sex + race + BMI + random effect per participant. Beta coefficients on the y-axes come from diet-stratified linear models of the structure: outcome ~ $\log_2$ fold change metabolite + pre chamber $\log_2$ metabolite level + age + sex + race + BMI.

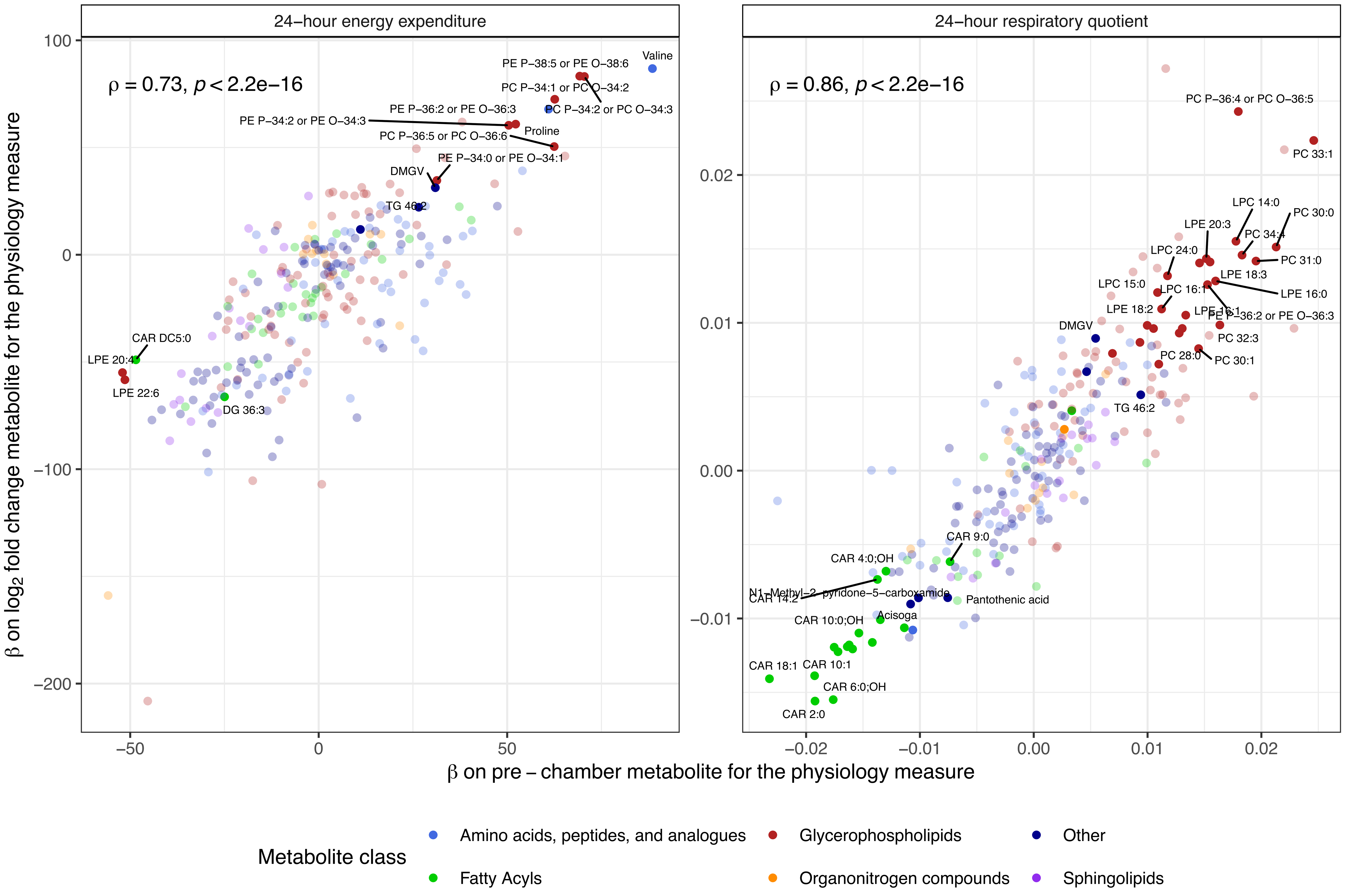

Supplemental Figure 9: Comparison of effects of pre-chamber metabolite levels and $\log_2$ fold change metabolite on physiologic measures. Scatterplots comparing beta coefficients from the pre-chamber metabolite variable and the $\log_2$ fold change metabolite variable for linear mixed models. Points are faded if neither beta coefficient had an FDR<5%.

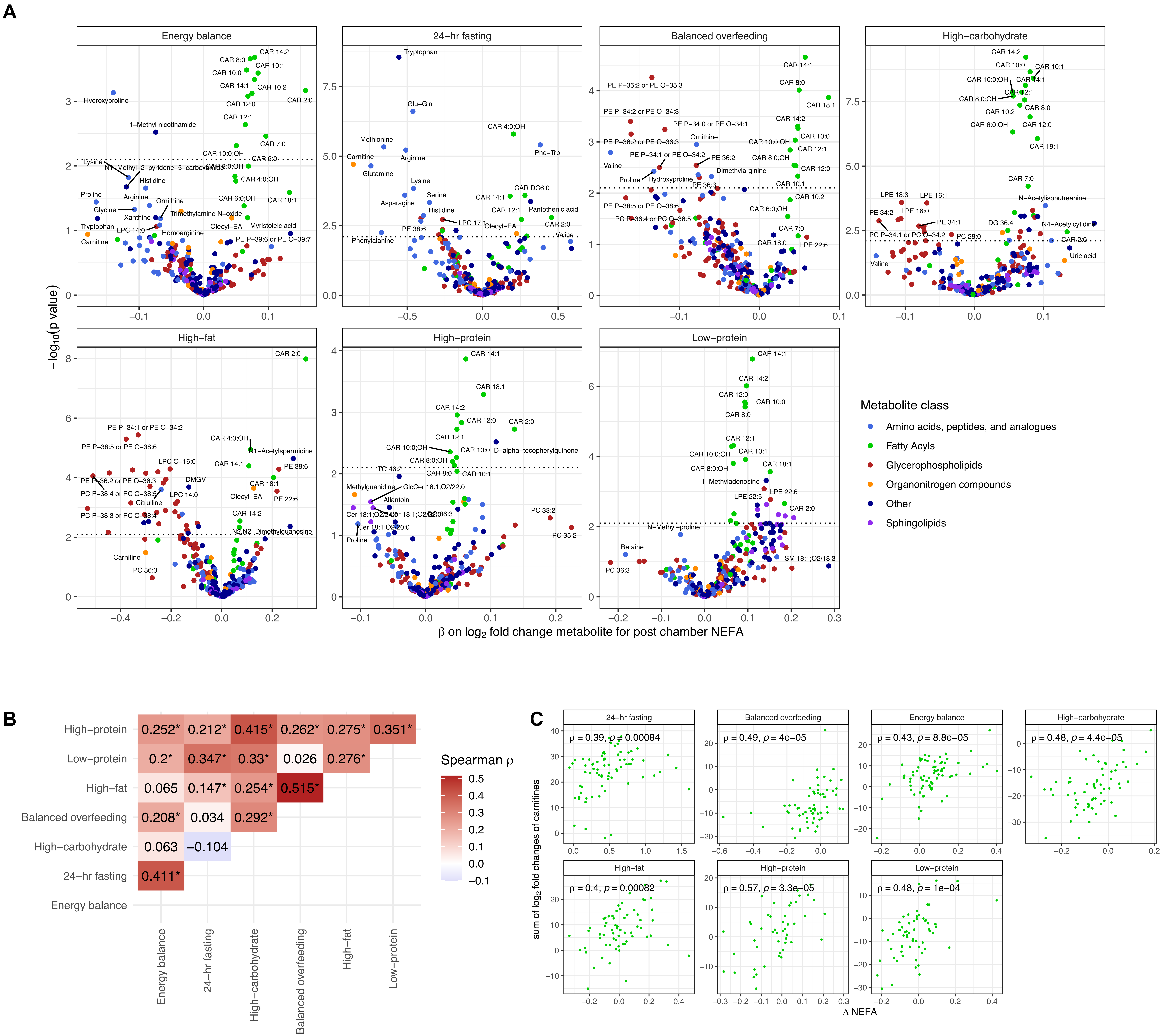

Supplemental Figure 10: Relations between log2 fold change metabolite and change in non-esterified free fatty acids (NEFA) in diet-stratified models. (**A**) Diet stratified models for NEFA with beta coefficients on the log2 fold change metabolite. (**B**) Spearman correlation heatmap of the beta coefficient on log2 fold change metabolite on the outcome of post-chamber NEFA (adjusted for pre-chamber NEFA and pre chamber metabolite and age/sex/race/BMI) across dietary chambers. (**C**) Supplemental Figure 7: Correlation between the sum of log2 fold changes in carnitines within participants correlated against the change in NEFA.

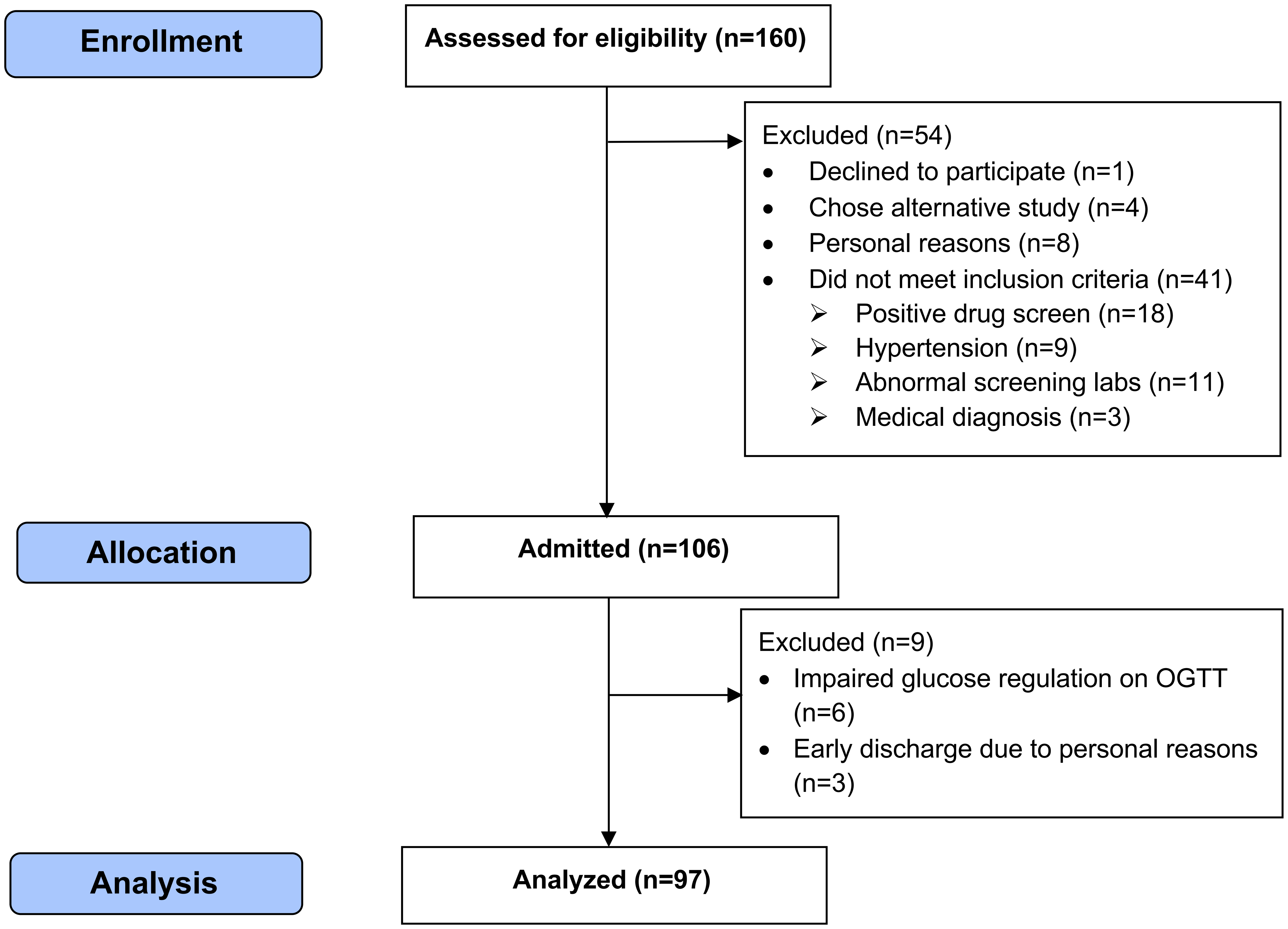

Supplemental Figure 11: CONSORT diagram for study.
